# Viscoelastic Properties of Fully Biomass-Based Transparent Plastic Comprising Cellulose Acetate and Citrate Ester

**DOI:** 10.3390/ma15093038

**Published:** 2022-04-22

**Authors:** Takeyoshi Kimura, Takumitsu Kida, Masayuki Yamaguchi

**Affiliations:** School of Materials Science, Japan Advanced Institute of Science and Technology, 1-1 Asahidai, Nomi 923-1292, Japan; s2120012@jaist.ac.jp (T.K.); tkida@jaist.ac.jp (T.K.)

**Keywords:** cellulose acetate, triethyl citrate, viscoelastic properties, rheology, elongational viscosity

## Abstract

Viscoelastic properties including melt processability were evaluated for a fully biomass-based glassy plastic comprising cellulose acetate (CA) and triethyl citrate (TEC). The TEC exerted an excellent plasticizing effect without dissolving the CA crystals. Pure CA has poor melt processability. In contrast, the TEC-plasticized CA had good melt-processability at 205 °C, which is lower than the degradation temperature of CA. Extrusion was possible even at 1000 s^−1^ without any flow instabilities, similar to conventional plastics showing good processability at extrusion. Furthermore, there was marked strain-hardening behavior in the transient elongational viscosity, suggesting that various processing operations are possible, such as a long-chain branched polymer. This biomass-based plastic can be used as a substitute for conventional glassy plastics because it is highly transparent and its softening temperature is above 100 °C.

## 1. Introduction

As concern for environmental issues increases, much attention has been focused on cellulose-derived materials. Among these materials, cellulose acetate (CA), which is produced by the acetylation of cellulose, is one of the most well-known [1,2,3]. CA has high heat resistance, good mechanical properties, and excellent transparency. However, it would have wider applicability and could be used as a substitution for conventional plastics if it had better melt processability. This problem is due to its low degradation temperature, which is almost the same as its melting point [4,5,6]. It is possible to melt-process low-molecular-weight cellulose acetates after adding a plasticizer, although they have poor mechanical properties. Propionyl and butyryl groups are more effective at decreasing the crystallinity of cellulose acetates and are currently available as melt-processable cellulose acetate propionate (CAP) and cellulose acetate butyrate (CAB) [3,7]. However, they are not used widely owing to their poor cost performance. Therefore, a high-molecular-weight, melt-processable cellulose acetate would be highly desirable because it would be cost effective and would have excellent mechanical and optical properties.

Kamide and Saito [4] studied the relationship between the degree of acetyl substitution (D_S_) and the thermal properties of cellulose acetates, such as their melting points and degradation temperatures. They confirmed that the degradation temperature is lower than the melting point except for in cellulose acetates with a D_S_ of approximately 2.5. Furthermore, cellulose acetates with a D_S_ of 2.2–2.5 have low degrees of crystallinity [5,8], suggesting that they also have good melt processability [9,10]. However, these cellulose acetates have high viscosity, even when their molecular weight is low. This is due to their high glass transition temperatures and the presence of crystals that act as pseudo-crosslink points. Therefore, the addition of a plasticizer is usually required for melt processing using conventional machines. However, there have only been a few studies on the rheological properties of plasticized cellulose acetates, especially those containing a biomass-based plasticizer.

Herein, we used a cellulose acetate with a Ds of 2.18 that has good biodegradability [3]. Citrate esters were used as biomass-based plasticizers because they are thermodynamically miscible with cellulose acetates [9,11,12,13,14,15,16]. The plasticizer molecules are known to be incorporated into polymer chains of cellulose acetate. In this study, the rheological properties in both solid and molten states were evaluated considering the role of crystals. In particular, the species and content of citrate esters were determined to show good melt processability similar to conventional plastics. Although the crystals in the cellulose acetates did not affect their transparency, they had a marked impact on their deformation behaviors during hot-stretching to prepare optical films [6,17]. Therefore, rheological responses under elongational flow were also evaluated in the present study.

## 2. Materials and Methods

### 2.1. Samples and Film Preparation

The polymeric material used in the present study was a commercially available CA provided by Daicel Corp. (Osaka, Japan). Its Ds was 2.18, and its number- and weight-average molecular weights determined with poly(methyl methacrylate) standards were 7.4 × 10^4^ and 2.1 × 10^5^, respectively. Commercially available plasticizers that show good miscibility with CA [11,12,13,14,15,16], such as triethyl citrate (TEC), tributyl citrate (TBC), and tributyl *o*-acetylcitrate (TBAC), were provided by Jungbunzlauer Suisse AG (Basel, Switzerland), and were used without further purification.

The films were prepared by a solution-casting method as follows. CA and one of citrate esters were dissolved in a mixed solvent comprising dichloromethane (CH_2_Cl_2_) and methanol (CH_3_OH) in a 9-to-1 weight ratio and stirred for 24 h at 25 °C. Solutions with 4 wt% CA with/without a citrate ester were poured onto glass plates and left overnight to allow the solvent to evaporate, then vacuum dried at 80 °C for 3 h. The contents of the citrate esters were 10, 20, and 40 wt%. The obtained samples were compressed into flat films (500 μm thick) using a compression molding machine at 190 °C (for the samples containing 20 or 40 wt% citrate ester) and 230 °C (for the other samples) at a pressure of 20 MPa for 3 min, and subsequently cooled in another compression-molding machine at 25 °C. The obtained 500 µm thick films containing 40 wt% TEC did not show optical retardation, which was confirmed by crossed polars, suggesting that they were free from the residual stress. In the case of the films with 20 wt% of a plasticizer, however, optical retardation was detected slightly. The films were stored in a temperature- and humidity-controlled chamber at 25 °C and 50% relative humidity for 1 day before obtaining the measurements.

### 2.2. Measurements

Wide-angle X-ray diffraction (WAXD) patterns were collected with a graphite monochromatized CuKα radiation beam using an R-AXIS IIc flat imaging plate detector (Rigaku, Akishima, Japan). The films were exposed for 6 min per shot by directing the X-ray beam normal to the film plane.

The temperature dependencies of the tensile storage modulus *E*’ and loss modulus *E*” between 25 and 260 °C were determined using a Rheogel-E4000 dynamic mechanical analyzer (UBM, Mukou, Japan). The frequency and heating rate were 10 Hz and 2 °C/min, respectively. Rectangular films (4 mm wide and 15 mm long) were used.

The angular frequency dependencies of the shear storage modulus *G*’ and loss modulus *G*” were evaluated using an AR2000ex rotational rheometer (TA Instruments, New Castle, DE, USA) with a 25 mm*f* parallel-plate geometry. The measurements were obtained at 205 °C. The angular velocity was in the range 628.3–0.01 rad/s.

The transient uniaxial elongational viscosity was evaluated using the rotational rheometer equipped with an SEG2-G universal testing platform (Xpansion Instruments, Tallmadge, OH, USA) at 205 °C. Rectangular specimens (10 mm wide, 17 mm long, and 0.5 mm thick) were used.

The steady-state shear viscosity *h* was measured using a 140 SAS-2002 capillary viscometer (Yasuda Seiki Seisakusho, Nishinomiya, Japan) at 205 °C and 215 °C. Two cylindrical dies with length (mm)/diameter (mm) (*L*/*D*) values of 10/1 and 20/2 were used. The entrance angles of both dies were 180°.

## 3. Results and Discussion

**Figure 1:**
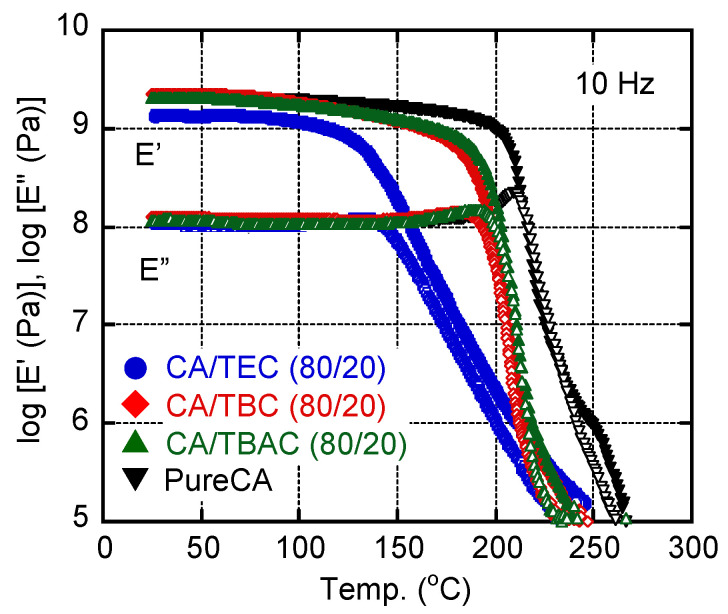
Temperature dependencies of tensile storage modulus *E*’ (filled symbols) and loss modulus *E*” (open symbols) at 10 Hz of CA films with 20 wt% TEC (circles), 20 wt% TBC (diamonds), and 20 wt% TABC (triangles). Data for the pure CA film were also plotted as inverted triangles.

All the films containing 20 wt% citrate esters were transparent, as was the pure CA film. The dynamic mechanical properties were initially evaluated using these films. Figure 1 shows the temperature dependencies of the tensile storage modulus *E*’ and loss modulus *E*” at 10 Hz. Glassy and transition regions are clearly discernable in the figure. The glass transition temperature *T_g_* of the film with TEC was significantly lower than the glass transition temperatures of the other films, although TEC has a short alkyl chain. The results demonstrate that TEC has the strongest plasticizing effect for CA among the citrate esters used. However, the slope of *E*’ for the film with TEC was not steep and featured a weak and broad *E*” peak, suggesting a broad relaxation mode during glass-to-rubber transition. Although these results seem to be contradictory, they must be attributed to the crystals in the CA. Because TEC cannot dissolve CA crystals, as shown later, the system has pseudo-crosslink points that trap amorphous chains and restrict segmental motions as discussed by deGennes [18]. As a result, the dynamic mechanical properties, including the relaxation mode during glass-to-rubber transition, were greatly affected. Correspondingly, the slope of *E*’ beyond the *T_g_* was not steep for CA/TEC (80/20). Both TBC and TBAC are also incapable of dissolving CA crystals, but TEC has the best plasticizing effect. The *E*’ values at high temperature would be determined by the entanglement density and crystallinity that act as crosslink points as demonstrated by Bendaoud and Chalamet [19] and Dreux et al. [20]. It was found that all the plasticized films had similar *E*’ values at approximately 230 °C owing to crystals, suggesting that the melting point existed around this temperature.

Hereafter, we used TEC as a plasticizer and evaluated the effect of the amount of TEC on the rheological properties of the CA in the solid and molten states. First, we confirmed there was no bleeding out on the surface of the film containing 40 wt% TEC, even after 1 month at 25 °C. Furthermore, we prepared another CA film with 70 wt% TEC and confirmed that the film was transparent without bleeding out.

**Figure 2:**
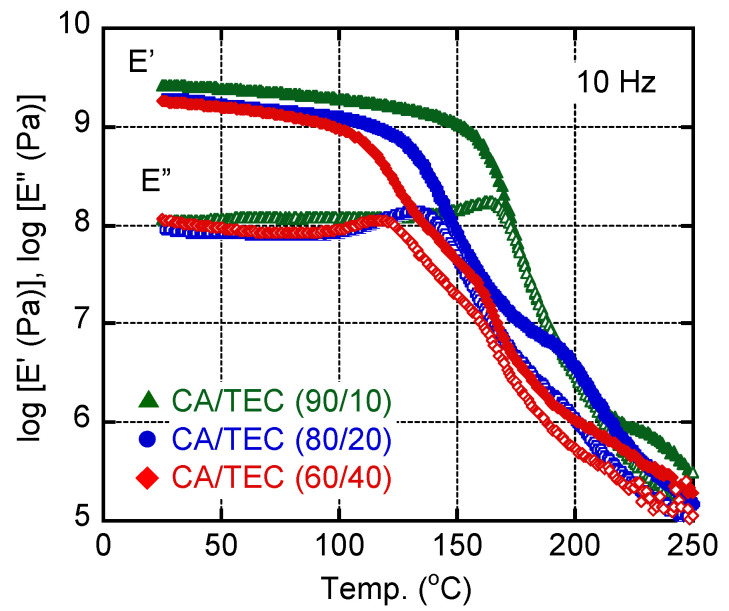
Temperature dependencies of the tensile storage modulus *E*’ (filled symbols) and loss modulus *E*” (closed symbols) at 10 Hz of CA films with various amounts of TEC: 10 wt% (triangles), 20 wt% (circles), and 40 wt% (diamonds).

Figure 2 shows the dynamic tensile moduli of films containing various amounts of TEC. Although the moduli dropped at low temperatures as the TEC content increased, the *E*’ values at approximately 250 °C were similar, i.e., 0.2–0.3 MPa, irrespective of the TEC content. This suggested that CA crystals existed in the film, even with 40 wt% TEC. The peak temperature of the *E*” curve of the film with 40 wt% TEC was approximately 120 °C. Moreover, the *E*’ values of the CA/TEC (60/40) film were higher than 100 MPa at temperatures below 150 °C. These results suggest that the softening temperature of this plasticized film must be higher than those of transparent conventional plastics, such as polystyrene, poly(vinyl chloride), and poly(methyl methacrylate).

**Figure 3:**
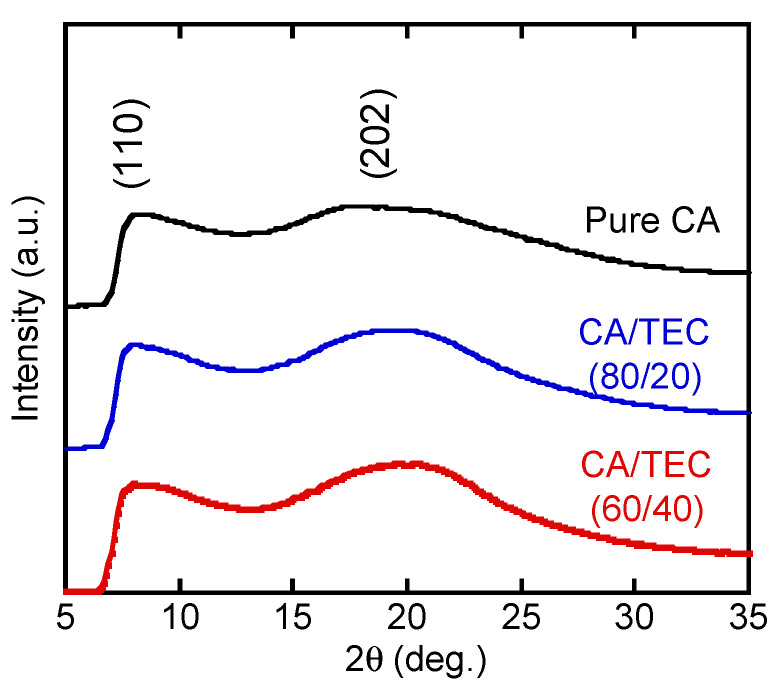
WAXD profiles of the CA and CA/TEC films. The numerals in brackets represent the Miller indices.

Because it was difficult to assess the crystallinity, which would be attributed to the degradation of CA, by differential scanning calorimetry, WAXD profiles were obtained to evaluate the crystallinity, as shown in Figure 3. Besides a broad amorphous hallow, there were two ambiguous peaks at 19.1° and 8°. They are ascribed to the (202) plane of typeⅠ and the (110) plane of type Ⅱ cellulose crystals (the numbers in brackets are the Miller indices) [21,22,23,24,25].

The results demonstrated that crystals existed in the films irrespective of the TEC content. The addition of TEC enhanced the crystallinity of CA because the crystalline diffraction peaks became obvious. This phenomenon is explained by the enhanced segmental mobility needed for crystallization. A similar phenomenon was reported for plasticized crystalline polymers with slow crystallization rates, such as poly(lactic acid) [26,27,28].

**Figure 4:**
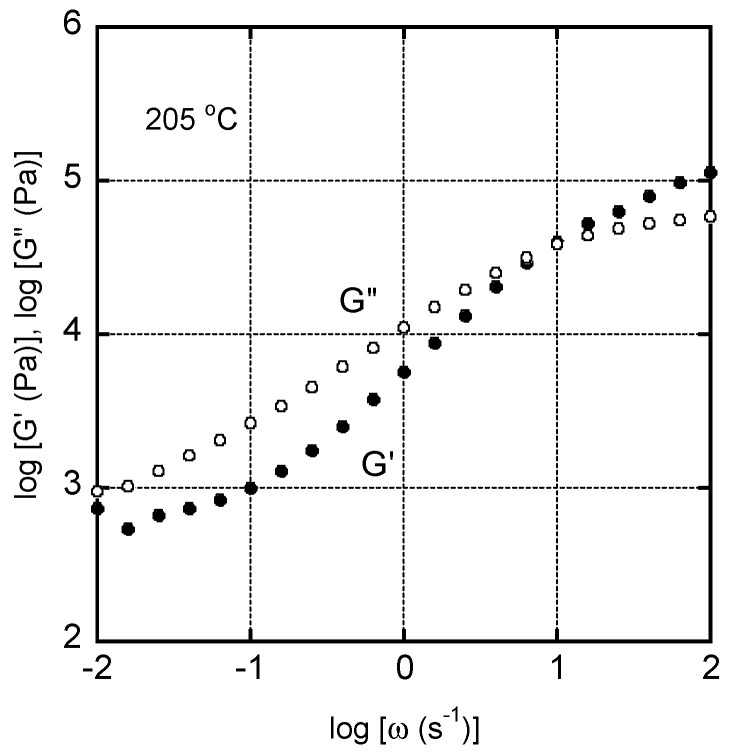
Angular frequency dependencies of shear storage modulus *G*’ and loss modulus *G*” of CA/TEC (60/40) at 205 °C.

The angular frequency dependency of the oscillatory shear modulus of CA/TEC (60/40) is shown in Figure 4. The measurements were performed from the high angular frequency and it took over 57 min for one measurement. The shear storage modulus *G*’ and loss modulus *G*” had similar values of approximately 10 s^−1^, indicating an average relaxation time of approximately 0.1 s. The *G*” values decreased monotonously with decreasing angular frequency in the experimental region. The slope was much less than 1, suggesting a long relaxation mode. Furthermore, there seemed to be a plateau in the shear storage modulus *G*’ in the low angular frequency region. The *G*’ value at the angular frequency of 0.01 s^−1^ increased slightly compared with the value at 0.0158 s^−1^. This must be attributed to crystal growth during the measurement [29,30]. Because the frequency sweep measurement was performed from the high frequency, the residence time measured at 0.01 s^−1^ was long, leading to crystallization. These results indicate that the system behaves like a long-chain branched polymer because of CA crystals during melt processing.

**Figure 5:**
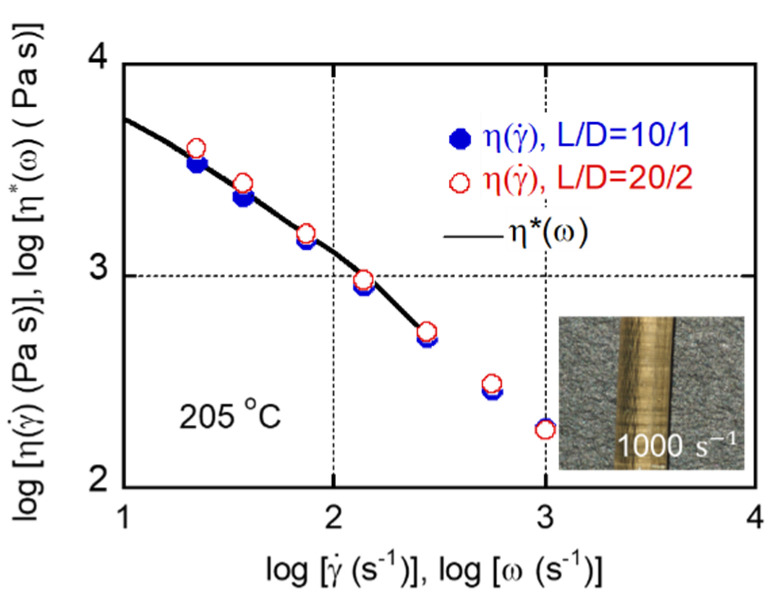
Steady-state shear viscosity $\eta \left(\dot{\gamma }\right)$ of CA/TEC (60/40) at 205 °C. The photograph shows a strand extruded at 1000 s^−1^ through a die with an *L/D* ratio of 10/1. The solid line represents the complex shear viscosity $\eta^{*}\left(\omega \right)$.

The steady-state shear viscosity $\eta \left(\dot{\gamma }\right)$ of CA/TEC (60/40) was evaluated using a capillary rheometer, as shown in Figure 5. Both shear rates and shear viscosities were the values at the wall without Bagley and Rabinowitsch corrections. The shear viscosity was in the range of standard grades of conventional melt-processable plastics such as polypropylene and polystyrene. Furthermore, the extruded strands were smooth, without any flow instabilities, even at 1000 s^−1^, as shown in the figure. These results demonstrate that most melt-processing operations, including injection molding, are possible with the plasticized CA. The figure comprises a plot of the data obtained using various circular dies with the same *L*/*D* ratio. The curves were almost the same, indicating that there was no slippage on the wall surface [31,32]. Moreover, the absolute values of the complex shear viscosity $\eta^{*}\left(\omega \right)$, calculated from the oscillatory shear moduli, were identical to the steady-state shear viscosity, demonstrating that the Cox–Merz relationship was applicable. These experimental results indicated that CA/TEC (60/40) behaves as a simple polymer melt with long-chain branches [33,34], not a network polymer. Most probably, the crystals act as branch points.

**Figure 6:**
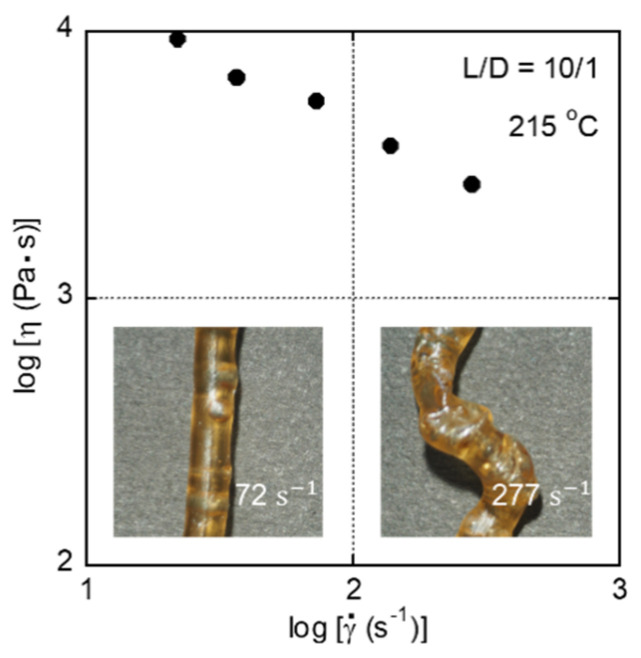
Shear viscosity of CA/TEC (80/20) at 215 °C. The photographs show strands extruded at 72 and 277 s^−1^ through a die with an *L*/*D* ratio of 10/1.

Capillary extrusion was also performed on CA/TEC (80/20). At 205 °C, however, severe melt fracture was detected, even at the lowest shear rate. Therefore, the extrusion was carried out at 215 °C. As shown in Figure 6, severe gross melt fracture, ascribed to a large elongational stress at the die entry [29,35,36], still appeared, even at 72 s^−1^. Presumably, a well-developed long chain branch structure, leading to a prolonged relaxation time, is responsible for this rheological behavior. This result corresponded with the residual stress in the film prepared by compression molding at 190 °C. Furthermore, the strands became yellow owing to the thermal degradation of the CA [37]. Therefore, melt processing at this temperature is not recommended.

The capillary extrusion measurements demonstrated that CA/TEC (60/40) is suitable for various melt-processing operations such as extrusion and injection. During some processing operations, including T-die processing, tubular film blowing, blow molding, thermoforming, and foaming, the rheological responses under elongational flow are very important. A molten polymer that exhibits strain hardening behavior in the transient elongational viscosity has reduced neck-in levels during T-die processing [38], a stable bubble during tubular film blowing [39], uniform product thickness following thermoforming and blow molding [40], and fine cell structure during foaming [41].

**Figure 7:**
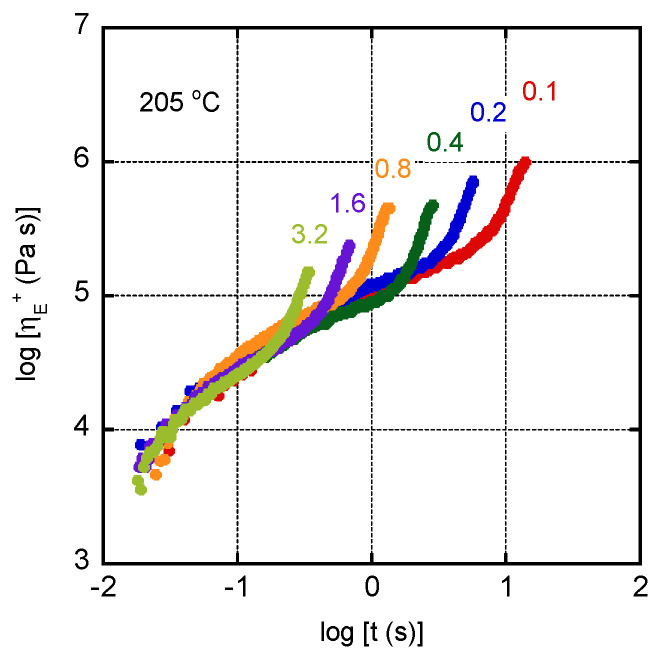
Growth curves of elongational viscosity $\eta_{E}{}^{+}$ of CA/TEC (60/40) at 205 °C. The numerals in the figure represent the strain rates (s^−1^).

The growth curves of uniaxial elongational viscosity $\eta_{E}{}^{+}$ of CA/TEC (60/40) are shown in Figure 7. The measurements were performed at 205 °C. Strain-hardening behavior, i.e., rapid growth of the elongational viscosity, was obvious at all strain rates, which is typical of long-chain branched polymers [36,38]. Considering that most conventional plastics without long-chain branches hardly show strain-hardening behavior, this must be attractive.

The strain hardening must be attributed to the crystals in the polymer, which act as branch points.

## 4. Conclusions

The viscoelastic properties of fully biomass-based transparent plastics, i.e., CA plasticized by citrate esters, were studied in both solid and molten states. Among the citrate esters employed, TEC had a good plasticizing effect, although it does not dissolve CA crystals. Various melt-processing operations were possible without yellowing at 205 °C, i.e., lower than the degradation temperature of CA, by the addition of 40 wt% TEC. Although the film contained a large amount of TEC, there was no bleeding out, even after 1 month. Furthermore, the softening temperature of CA/TEC (60/40) was higher than those of conventional transparent plastics such as polystyrene, poly(vinyl chloride), and poly(methyl methacrylate).

It was possible to process CA/TEC (60/40) by capillary extrusion without any flow instabilities, even at 1000 s^−1^, demonstrating that the sample shows good processability at extrusion even compared with conventional commodity plastics. Furthermore, the CA crystals play an important role in the rheological response under elongational flow. Because crystals act as pseudo branch points at processing temperature, the sample exhibited marked strain hardening during the transient elongational viscosity phase. These rheological properties are typical ones of long-chain branched polymers. This resulted in favorable processability at film processing, blow-molding, and thermoforming. Because of the excellent melt processability at a relatively high softening temperature, this biomass-based plastic can be employed instead of commodity plastics to meet the global demands.

## Data Availability

Not applicable.
